# Supplementary material for: Biological age estimation using circulating blood biomarkers
Source: Commun Biol. 2023 Oct 26;6:1089. doi: 10.1038/s42003-023-05456-z (PMC10603148; doi:10.1038/s42003-023-05456-z)
Supplement: Supplementary file 3 — Description of Additional Supplementary Files [file 42003_2023_5456_MOESM3_ESM.pdf]

Description of Additional Supplementary Files

File name: Supplementary Data
Description: Excel file with multiple tabs containing the source data behind the graphs in the manuscript.
